# Recent advances for cancer detection and treatment by microfluidic technology, review and update

**DOI:** 10.1186/s12575-022-00166-y

**Published:** 2022-04-28

**Authors:** Nasrin Bargahi, Samaneh Ghasemali, Samaneh Jahandar-Lashaki, Atefeh Nazari

**Affiliations:** 1 Student Research Committee, Tabriz University of Medical Sciences, Tabriz, Iran; 2 Biotechnology Research Center, Tabriz University of Medical Sciences, Tabriz, Iran; 3 Drug Applied Research Center, Tabriz University of Medical Sciences, Tabriz, Iran

**Keywords:** Microfluidic, Laminar flow, Tumour, Metastasis

## Abstract

Numerous cancer-associated deaths are owing to a lack of effective diagnostic and therapeutic approaches. Microfluidic systems for analyzing a low volume of samples offer a precise, quick, and user-friendly technique for cancer diagnosis and treatment. Microfluidic devices can detect many cancer-diagnostic factors from biological fluids and also generate appropriate nanoparticles for drug delivery. Thus, microfluidics may be valuable in the cancer field due to its high sensitivity, high throughput, and low cost. In the present article, we aim to review recent achievements in the application of microfluidic systems for the diagnosis and treatment of various cancers. Although microfluidic platforms are not yet used in the clinic, they are expected to become the main technology for cancer diagnosis and treatment. Microfluidic systems are proving to be more sensitive and accurate for the detection of cancer biomarkers and therapeutic strategies than common assays. Microfluidic lab-on-a-chip platforms have shown remarkable potential in the designing of novel procedures for cancer detection, therapy, and disease follow-up as well as the development of new drug delivery systems for cancer treatment.

## Introduction

One of the world's main causes of mortality is still cancer. In 2016, only in the United States, about 1.6 million newly diagnosed cases of cancer and roughly 500,000 cancer-related deaths were recorded [1, 2]. Solid tumours that form in the tissues of the lung, breast, bronchus, rectum, colon, prostate, and bladder are the most prevalent types of cancer, as reported by the American Cancer Society [2]. According to estimates, more than 15 million people in the United States have been diagnosed with cancer, which is predicted to increase to more than 19 million by the year 2024, leading to a huge financial burden of more than 130 billion dollars a year [1, 2]. Although new technologies have made it normal to detect solid tumors, more advanced technologies are required to detect cancers before the onset of symptoms, track disease progression and assess treatment response in patients in order to enhance prognosis, improve life quality, reduce medical costs and increase the chances of recovery in cancer patients [3]. Microfluidic technology makes it possible to manipulate fluid on a micron-scale from 1 to 1,000 µm, about the size of a single cell [4, 5]. The microfluidic platform has proven to be a powerful tool for performing complicated, costly, and time-consuming as well as challenging laboratory techniques on a microchip that solves these problems. Due to its micron-scale size, microfluidics technology can execute intricate operations quickly and with a low reagent level. Consequently, it's a highly efficient platform for diagnosing due to its high capacity, time-saving features, and precision. To achieve high levels of accuracy that are not feasible with traditional macro-scale techniques, micro-scale devices can be developed to tackle the problem while also guaranteeing that every single cell or biomolecule is assessed homogeneously, which is critical when exploring low-abundance cells and biomolecules. Microfluidics can also be used to create completely original separation strategies by using scaling effects to generate laminar flow, boost secondary forces, and outline special geometries to specifically navigate, confine, and collect cells and cell-derived products [6–9]. Microfluidic methods for cancer diagnostics, such as detecting cancer cells and cell-derived products, have sparked a lot of attention so far. According to the reagents, any fluid flowing through a microfluidic chip experiences laminar flow due to the platform scale. To meet the specific needs of the user, this flow can be adjusted and controlled with high precision [10]. Microfluidic tools have also gotten attention as a potent platform for therapeutic target discovery and treatment response monitoring, enabling intricate interplay in the metastatic microenvironment and manipulation of individual factors [11]. The most critical point is that the microfluidic device allows the extraction of individual tumor cells of interest. It also facilitates genomics, transcriptomics, and even metabolomics, as well as allows the identification of individual cell clonal backgrounds. As a result, the platform can be used with a large-scale impartial omics approach [12]. For catching rare cells, several microfluidic technologies have been developed, such as circulating tumor cells (CTCs), circulating fetal cells, and stem cells [13]. One of the frequent and severe consequences of cancer is metastasis or the migration of cancer cells. To appreciate the significance of investigating specific processes and treatment options for metastases, keep in mind that tumor metastasis causes more than 90% of cancer deaths [14]. Due to the possibility of including complex interactions in the microenvironment of metastatic cells and altering individual components, microfluidic technology has developed as a potent platform for drug target discovery and therapeutic response evaluation [3]. The system's only known limitation is the lack of a suitable microfluidic device that executes any laboratory procedure on a single chip. At present, no chip analyses and displays information in a way that anyone can understand, but some chips can perform several processes simultaneously, such as isolation and identification. It is possible to diagnose cancer before it progresses and save patients' lives by reducing and eliminating the limitations on this technology's diagnosis capabilities [15]. This paper is structured as follows. Section 1 serves as an introduction. Section 2 presents a brief overview of the microfluidic lab-on-a-chip platforms with an emphasis on the gap of a standard method for constructing a microfluidic system. Section 3 describes the challenges of replacing chip-in-a-lab with lab-on-a-chip. In the next section, the related works done for in vitro cancer detection by microfluidic technologies are discussed. In Sect. 5, we describe microfluidics and novel lab-on-a-chip applications that have the potential to induce microenvironmental tumor signals, for studying the dynamic microenvironments of tumor cells. The microfluidic approaches to the study of angiogenesis and anti-angiogenesis are highlighted in Sect. 6. Diverse microfluidics strategies for the study of cancer metastasis is reviewed in Sect. 7. In Sect. 8, recent advances in microfluidic device technology are discussed with a focus on mimicking the crucial functions of tissues and organs to investigate the interaction between cancer and other organs. Cost-effective and high throughput platforms for studying cancer therapeutics such as drug screening, drug delivery, and drug formulation are presented in Sect. 9. Section 10, gives a number of the advantages and challenges of microfluidic devices in fabrication and application.

## Overview on microfluidic lab-on-a-chip platforms

Microfluidic research dates back to the early 1990s and is currently advancing at a rapid pace. The global microfluidic market was estimated to be worth around $2.5 billion in 2016 and $10.06 billion in 2018, according to Grand View Research and Markets and Markets. A study by Grand View Research and Markets found that the marketplace will continue to expand at a compound annual growth rate (CAGR) of 18.4 percent and 22.6 percent between now and 2023. The report states that by 2023, the market will be worth USD 27.91 billion [16]. A microfluidic device is a collection of fluidic operating units developed to be seamlessly integrated into a specific fabrication process. A standard method for minimizing, integrating, automating, and parallelizing chemical (biological) processes can be provided by a microfluidic platform. Thousands of scientists have strived hard over the last thirty years to develop micropumps [17–20], microvalves [21], micromixers [22], and other microfluidic liquid manipulating tools. However, one of the most significant requirements for the effective design of lab-on-a-chip technology, which is a coherent manufacturing and interfacing system, often does not yet exist. This gap can be filled only by constructing a microfluidic system that provides a quick and simple application of biochemical protocols according to conventional components. The concept was inspired by the significant influence of platforms in the business of application-specific integrated circuits (ASIC), where established components and procedures allowed for quicker design and cost-effective manufacture of electronic circuitries. To apply this to microfluidic technology, you'll require a set of tested microfluidic elements, each capable of performing a single step or function of the main fluid control unit. Fluid metering, fluid transport, fluid mixing, piping, and molecule or fragment isolation or centralization are only a few of the fundamental unit applications that are used in the protocols of laboratories. For the easy performance of program-specific measurements on the microfluidic platform, a sufficient number of microfluidic unit operations must be provided that can be easily combined [23]. Microfluidic devices are more in demand than conventionally sized devices due to less waste of energy and time, greater flexibility [24], less sample and reagent use [25], lower production and conductivity expenses [26] and retention of traditional features such as rapid sample analysis [25], automation, high-resolution and high-efficiency screening [27]. The number of publications devoted to the implementation and development of novel microdevices has also increased, indicating that microfluid technology is becoming more popular. The groundbreaking micro or nanofabrication methods developed using various soft lithography techniques allowed microfluidic platforms to evolve [28, 29]. These techniques enable the fabrication of physical items at the micro or nanometer scale [30]. A microfluidic device typically consists of channels [31], chambers [32] and other structures such as pillars [33], rods, wires and tubes fabricated at the nanoscale [34, 35]. The integration of these nano-scale parts into a single microfluidic platform allows for real-time monitoring of cancer cell behaviors in response to a range of events and stimuli [16].

Standards in the microfluidic sector have yet to be developed, leading to the combination of tools from other areas, such as smartphones for displaying output signals [36], microscope glass slides, microtiter-plates, and Luer connections [37]. As a result, putting them together in a packed system is complicated, which commonly takes the form of a self-titled "spider web assembly" in the laboratory, in which many tubes and wires attach the different pieces to one another. Furthermore, the microfluidic assembly favors a broad range of substrate elements, such as silicon, glass, and polymers like polydimethylsiloxane (PDMS). PDMS is a popular material among investigators, but it is deprived of the ability to be manufactured in high numbers with minimal expenditure, as well as of an extended shelf-life, resulting in the unsuitability of profitable production [38, 39]. A standardized platform is required to economically benefit from the microfluidic devices, which are fabricated from a variety of materials and have already been introduced in academia. A vast association of key industrial and academic partners has developed standardized elements that ensure adaptability and interworking between diverse system aspects to ensure successful system integration. The majority of the standards are explained in the contract of ISO 23:2016 workshop and command-paper [40, 41] which relates to footmarks and interlink situations for more miniature microfluidic parts [41]. Analytics is a key subject in microfluidic implementation. A number of biomolecules, comprised of proteins and nucleic acids, can be used as the examined molecule (analyte). Sufficient combining procedures, as well as very accurate fluid measurement and liquid control, are required to provide precise quantitative findings. In addition, for the performance of sophisticated analytical protocols, mechanization, mobility, wearability, and a vast variety of unit operations, are necessary. Cellular assays are the most complicated experiments to develop because the cells must be kept in a suitable environment to sustain their viability and activity (controlling pH, O2, CO2, nutrients, and so on). Cellular assays are essential for determining the influence of novel pharmacological entities on bioavailability, mutagenicity, toxicity, and undesired side-effects at various dosage concentrations. The design of single-cell analysis assays is the most exciting opportunity [42, 43]. High-throughput solutions and low usage of reagents in each test are prerequisites for cell-based tests [23].

## From chip-in-a-lab to lab-on-a-chip:

The field of lab-on-a-chip research and technology has seen major technological leaps toward sample handling, sample preparation, and sensing for use in molecular diagnostic devices. Despite this, the potential for developing practical point-of-care tools based on a lab-on-a-chip approach is limited to a fraction of the anticipated possibilities [44]. Almost three decades have passed since the first microfluidic publicity, which followed the advent of micro techniques for total analysis. Microfluidics is still struggling to gain across-the-board market penetration [45, 46]. This issue could be due to a lack of standards, concentration, and contact between scientific and industrial settings. Although several lab-on-a-chip systems have been demonstrated in the literature, the phrase chip-in-a-lab is more appropriate for the current microfluidic subject. Even if a limited number of leading commercial devices [such as the Abbott i-STAT and DNA polymerase chain reaction (PCR) machines] exist, the majority of lab-on-a-chip tools are still locked at technology readiness level (TRL) 3 or 4. At present, the majority of microfluidic technologies are not compact because system integration matters [47]. Although assistive devices are aspects of microfluid settings, they are bulky and difficult to downsize because they were primarily designed for other applications [38]. In addition, commercialization is difficult because the liquid control, display, and signal recognition elements are incompatible [47, 48]. These large assistive devices should be downsized to achieve accurate point-of-care (POC) microfluidic tools. Improving mobility and decreasing platform reliability will require minimizing and standardizing the footprint of assistive devices. It will also increase the integrity and compactness of the system, keeping microfluidic strategies from falling into the deadly valley that exists between academics and industry [36]. Scientists bridge the gap between the lab and the factory by supplying easy-to-manufacture modular building blocks that are compatible with mass production. These uniform blocks are used in conjunction with a fluidic circuit board that is tailored to the application. Standardized reusable parts reduce the time spent on design and fabrication, which reduces the time-to-market for industrial development. The standard system allows for the integration of multiple components into a single system. According to the researchers, this standardized platform fills the gap between "chip-in-a-lab" and "lab-on-a-chip" [41].

## Microfluidic technologies for in vitro cancer detection

### Circulating tumor cells

Circulating tumor cells (CTCs) enter the bloodstream from both primary and metastatic lesions, and they carry significant signatures of cancer progression and metastasis. These cells have a short half-life and can hold significant information for the detection, characterization, and monitoring of cancer [49]. The ability to capture and retain these rare cells from the bloodstream of cancer patients with high efficiency and purity is a major challenge in the analysis of CTCs as a biomarker in prognosis. Various methods, including molecular identification, immunocytological assays, enzyme-linked immunosorbent assay (ELISA), comparative genomic hybridization, functional characterization, and fluorescence in situ hybridization, have been recently explored to detect CTCs from patient blood. However, these techniques are time-consuming and require skilled operators and high-tech instrumentation [50]. Size-based and affinity-based methods are commonly used for CTC isolation and enrichment [51]. The size-based isolation methods are based on the physical properties of the CTCs. These methods are simple, fast, and have a high efficiency that capitalizes upon size differences between CTCs and non-tumor cells and is currently used for CTC detection [52]. Due to the separation of unmodified and unlabeled cells, membrane microfilters are mainly applied to isolate CTCs by size-dependent methods. A major problem with these filters is clogging by trapping larger cells or debris in the filtration, especially at a high flow rate and high cell density. In the cross-flow filtration process, the separation efficiency is increased by larger particles remaining in a suspended state, reducing the clogging problem [53]. The use of microfluidic technologies for the capture of rare CTCs offers the advantage of fast, reliable sample separation, resulting in excellent yields and high specificity of target cells. Accordingly, several methods use microfluidic devices, such as Aptamer-based separation, peptide-based detection, immunoaffinity purification, and cytosensor-based detection for the capture of CTCs from a patient’s blood [50].

#### Microfluidic aptasensors for CTCs detection

**Fig. 1:**
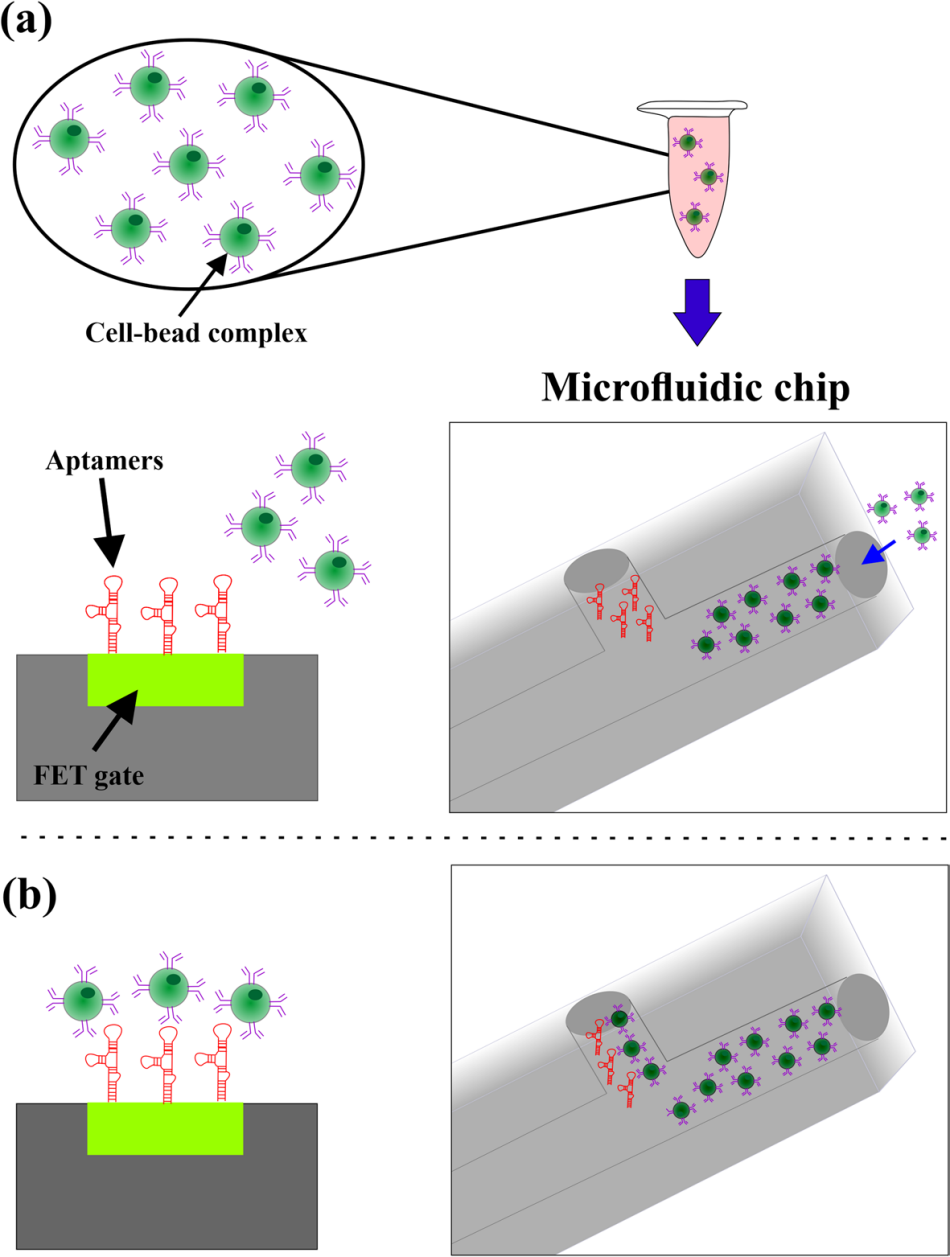
A schematic image of aptamer-based CTC capture and detection processes: (**a**) Sample injection; (**b**) CTCs trapping and FET sensing

Aptamers are one category of single-stranded oligonucleotides of DNA or RNA that have higher selectivity and stability than antibodies and can specifically bind to proteins, ions, or small molecules. They can detect a large number of analytes at a low cost and convert them into catalytically measurable signals [49, 54]. The various microfluidic aptamer-based biosensors are designed for CTCs detection. The gallium nitride (GaN) high electron mobility transistor (HEMT) based microfluidic aptasensor designed by Pulikkathodi et al. is based on a thermocurable polymer chip with fixed miniaturized sensors that detects and counts the amount of CTCs in the solution in conjunction with microfluid channels [55]. There is considerable interest in FET-based biosensors, among the many varieties of microfluidic aptasensors, for the direct use of non-labeled biological samples and the development of useful electrical signals [56]. These types of biosensors are capable of detecting and counting captured CTCs at physiological concentrations without the need for additional sample processing. Recently, Chen et al., using PDMS, have designed a novel FET-based aptasensor to increase the CTCs captured from the total blood sample, which could count up to 42 cancer cells (Fig. 1) [57]. In another study, Zhang and colleagues combined size-based microfluidics with surface-enhanced Raman spectroscopy (SERS) to detect biomarkers of breast cancer cell membranes and to easily diagnose breast cancer. For this purpose, CTCs were first isolated based on their size differences with blood cells, then, using different SERS aptamer vectors, markers on the cancer cells' membrane were simultaneously identified [58].

#### Microfluidic peptide-based biosensors for CTCs detection

Recently, sensor technologies based on peptides have been rapidly developed and can potentially be implemented in various clinical fields [59]. The use of screen-printed electrodes and peptides to design electrochemical biosensors and using nanomaterials to make microfluidic biosensors based on peptides, because of their unique electrical, optical, and structural properties and biocompatibility, has a high potential for detecting CTCs [50]. Hassanzadeh-Barforoushi et al. designed a microfluidic peptide-based biosensor and used a single-step fluorescent resonance transfer (FRET)-based biosensor that displayed the protease activity of a single cancer cell using the fluorescent signals produced by amino acid chain breakdown. The revolutionary method of microfluidic biosensors is able to detect CTCs in the circulatory system or in individual cancer cells in tumors [60].

#### Microfluidic immunosensors for CTCs detection

CTCs immunity-based techniques use the interaction between the antibody and the specific antigen, such as the epithelial cell adhesion molecule (EpCAM), which is an epithelial marker. EpCAM, a glycoprotein that is highly expressed on the surface of carcinoma cells, is one of the most reliable clinical biomarkers for CTC detection. A CTC-chip is an example of a microfluidic device making use of immunoaffinity purification. Microposts consisting of arrays of vertical cantilever beams made from flexible silicone and functionalized with anti-EpCAM are the basis for CTC-chips. In contrast to the other cells, when CTCs in the blood go through the microchannel, they attach to the antibody on the surface of the microposts and are captured [61, 62]. Bravo et al. developed an electrochemical microfluidic immunosensor using an AgNPs-PVA nanoplatforms to stabilize recombinant antibodies, including EpCAM, in the biological specimens. The EPCAM concentration was then determined via a reaction with HRP. The results showed this technique was faster than the ELISA kit used in clinical experiments [63]. Kumeria et al. designed a label-free reflectometric biosensor that was based on reflection interference spectroscopy (RIfS). In this technique, non-porous anodic aluminum oxide (AAO) is a platform for binding the biotinylated anti-EpCAM antibodies through streptavidin to it. Human cancer cells of epithelial origin specifically attach to anti-EpCAM antibodies and are detected without separation processes [64].

#### Microfluidic cytosensors for CTCs detection

The development of the nanotechnology field and the design of nanofibers, nanocolumns, nanotubes, etc. have led to the production of microporous nanofibers with various diameters. Such nanofibers can be a suitable environment for promoting cell adhesion and growth. Wu and colleagues developed a biosensor based on cells (cytosensor), which captured breast cancer cells (MCF7) with high EpCAM expression by creating three-dimensional bionic interfaces and cross-linking long nanofibers (glycolic acid-lactic acid) (PLGA) to the surface of Ni micropolar using an electrospinning method. Afterwards, through the use of EPCAM antibodies in a nanochip, MCF cells are electrochemically identified [65].

### Tumor cell derived exosomes

Exosomes are nanoscale vesicles comprised of lipids, proteins, mRNAs, micro RNAs (miRNAs), and other molecules that are formed by the process of endocytosis. They are released by most cell types into the extracellular space and are isolated from different body fluids [66]. Tumor-derived exosomes are of particular importance among biomarkers because of the substantial information contained in their molecular components and the role of these components in different stages of tumor formation and cancer development [67]. Isolation and purification of exosomes is the first step to their detection. At present, two isolation techniques based on exosomes’ physicochemical properties, including the immunoaffinity-based method and ultra-centrifugation (UC), are mainly used to purify target exosomes in bodily fluids. Immunoaffinity-based methods use antibody-coated magnetic beads for exosome isolation [68, 69]. The main disadvantages of these conventional methods, such as long separation times and complexity of sample processing, lead to the use of microfluidic techniques for the detection of exosomes in order to time-save and simplify steps of sample preparation [66]. Microfluidic technologies for the analysis of exosomes are mainly based on their physicochemical properties, such as size, electric charge, compressibility and deformability [70]. Active and passive microfluidic methods are two major categories for label-free exosome separation [71]. Passive techniques are mainly used for vesicle separation on the basis of differences in the inherent characteristics of the exosomes, such as size and electrical characteristics [72], whereas active microfluidic techniques, such as electroseparation and acoustofluidic, employ external fields and forces to induce exosome movement for their isolation and purification [73, 74]. The purified exosomes can be used for various purposes, such as drug delivery, disease diagnosis, precision therapeutics and tissue regeneration [75–78]. Lee and coworkers established an acoustic-based microfluidic chip that was designed for the separation of exosomes released from ovarian cancer cells into the culture. The installation of interactive digital converting electrodes in the center of the chip controlled the radiating force of the acoustic wave and adjusted the rate of sample flow. At the end of the chip, the various sizes of particles that had moved on the different sides of the flow were collected by diverse channels [73]. Research by Wu et al. showed that this chip could purify exosomes from total blood with an efficiency of 98.4% [79]. Liu and colleagues designed a novel digital detection technique based on immunoaffinity microfluidics for counting single exosomes in order to diagnose breast cancer. First, the immuno-magnetic beads were used to capture exosomes from the sample, and these exosomes were then connected to the fluorescent reaction enzyme by ExoELISA. Next, to form the microdroplets containing the most exosome, the magnetic bead complex was formed on the microdroplet chip, along with the fluorescent reaction substrate. Exosomes that were expressing distinct diagnostic biomarkers (GPC-1) were bound to enzymes that catalyzed the fluorescence of the substrate in the droplet. Finally, fluorescent droplets were counted by a digital detection method to detect exosomes [80]. Zhao et al. developed an ExoSearch chip for the isolation of exosomes from the plasma of ovarian cancer patients. Exosomes from plasma were stained with fluorescent dyes in order to detect exosomes and obtain multicolor fluorescence imaging. Such a chip has two fluid injection ports: one end is utilized for the sample, and the other end is utilized for the injection of a solution of immunomagnetic beads containing specific antibodies. In the serpentine channel, a Dean vortex and an inertia lift are created to amplify the complete mixing of the two currents and then absorbed through the magnetic field [81]. Kanwar and coworkers designed the microfluidic ExoChip in which the surface of its channel has been covered with a layer of biotinylated CD63 antibodies. Such a channel was used for capturing exosomes comprising antigens of CD63 from the supernatant of cell culture and serum of patients with pancreatic cancer. To quantify the gained exosomes, the lipophilic membrane fluorescent carbocyanine dye (Dio) was utilized [82].

### Cell-free tumor cell DNA and circulating RNA

**Fig. 2:**
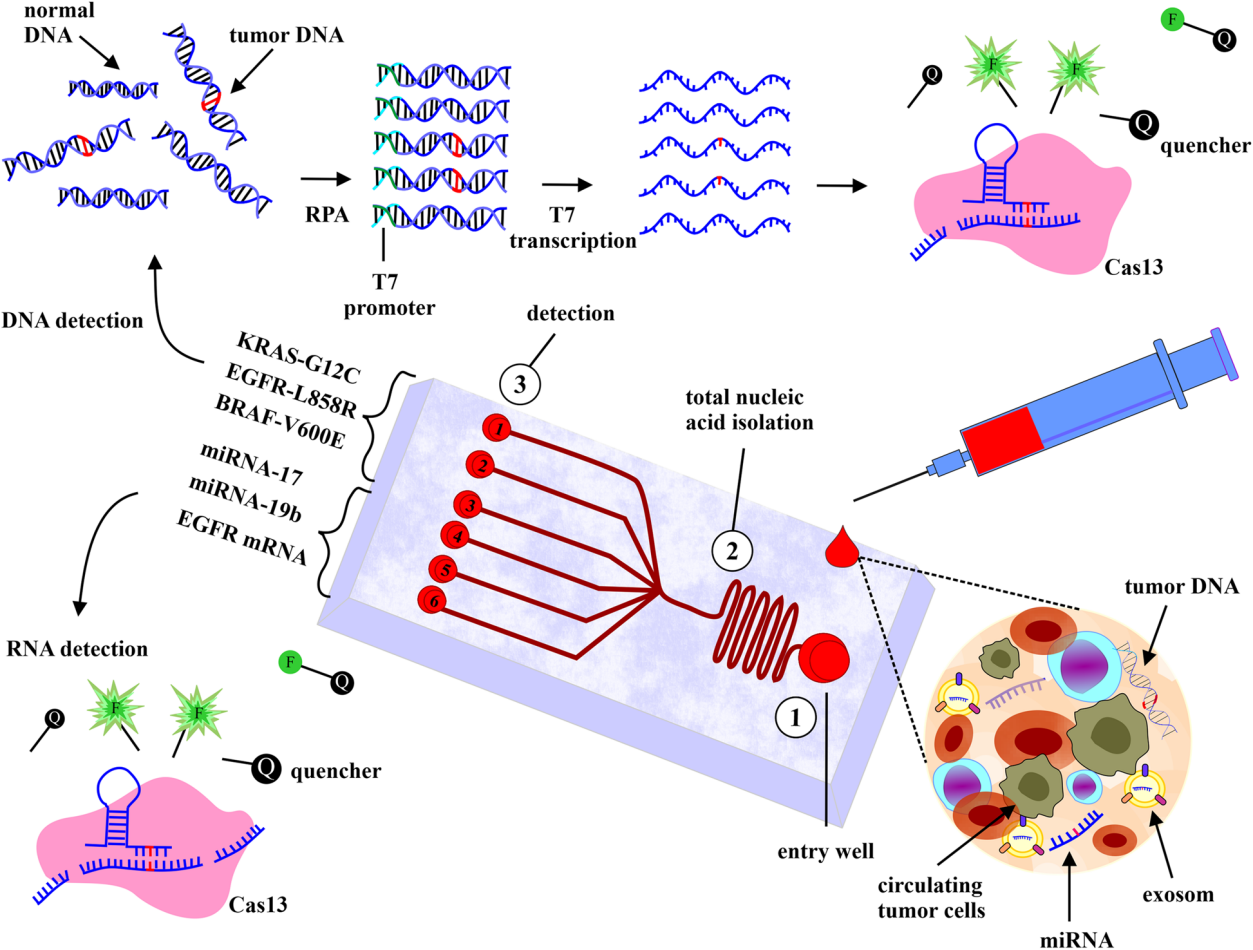
A hypothetical Cas13-based microfluidic biosensor for cancer diagnosis. First, the blood of NSCLC patients, containing materials derived from the tumor, enters directly into the entry well. After the isolation of the nucleic acids, they are followed into wells containing reagents for the detection of DNA mutations or quantifying overexpressed NSCLC-associated RNAs. Cas13 is activated by direct targeting of RNAs and subsequent matching of the target RNA sequence with the crRNA spacer sequences. This leads to cleavage of the target RNA and fluorescent reporter RNAs. On the other hand, after amplification of tumor DNAs with RPA, the T7 promoter sequence is added to the 5'-end of the RPA forward primer, and RPA amplicons are transcribed with T7. By binding to mutation-containing transcripts, Cas13 cleaves fluorescent reporter RNAs to provide the detectable signals

Circulating tumor DNA (ctDNA) is a sub-class of Cell-free DNA (cfDNA) that is released in the body fluids of diseased and healthy individuals. Removing cfDNA by DNases from the blood in healthy subjects happened within minutes. In cancer patients, the ctDNA accumulates in blood plasma and can be used to identify the disease as a prognostic and diagnostic biomarker [83]. ctDNA is more stable than CTCs and has a higher proportion in the bloodstream. Analysis of ctDNA as a tumor biomarker with high sensitivity by noninvasive liquid biopsy techniques is a requirement in personalized medicine [84]. Current techniques used for the analysis of ctDNA include whole genome or exome sequencing, next-generation sequencing (NGS), polymerase chain reaction (PCR), and emulsions, amplification and magnetic analysis (BEAMing) [85–87]. Although the detection of specific mutations associated with oncological diseases in ctDNA is more commonly used for different approaches such as early cancer diagnosis, cancer prognosis, and assessing treatment response, however, mutation analysis of ctDNA requires high-quality ctDNA extracts and highly efficient methods [84, 88]. In the past decade, the size and properties of ctDNA have led to increased attention to the use of microfluidic liquid biopsy devices for ctDNA detection. Because of the small volume of sample for microscale analysis and time-saving in microfluidic systems, microfluidic methods, such as the ctDNA concentration-based microfluidic channel, seven droplet-based digital PCR microfluidic system, dielectrophoretic capture, microfluidic multiplex PCR and microfluidic platform integrated with the Sanger sequencing method, are used for ctDNA detection [84]. Chaudhuri and coworkers established seven droplet-based digital PCR microfluidic methods for identifying somatic mutations through ctDNA detection. This system using TaqMan® probes isolates wild-type DNA and mutant DNA from each other with fluorescent signals. The KRAS oncogene can be detected accurately through this method. The limitation of this system is the number of droplets to analyze [89]. Bahga and colleagues designed electrodes for trapping dielectrophoretic on the device to isolate ctDNA. High-efficiency results were reported without specific details of the device's sensitivity level [90, 91]. Recently, Koboldt et al. designed a high-sensitivity microfluidic multiplex PCR sequencing technology to quantify ctDNA at low a concentration. To accomplish this, the multiplex PCR preamplification and sequencing techniques were integrated together, and the experiment was continued using the off-chip empirical Bayesian model to check for errors specific to specifying ctDNA. With such a method, the sensitivity and the specificity of ctDNA mutation detection in plasma from patients with pancreatic and ovarian cancer were 92% and 100%, respectively, matched to tumour tissues [92]. Campos et al. demonstrated a unique cheap plastic microfluidic surface using solid-phase microextraction (SPμE) to extract cfDNA. In microfluidics, clinical analysis was performed by extracting solid-phase using various techniques such as porous solid phase, immobilized magnetic beads, or micropillar structure. In 2018, Campos et al. utilized the array of micropillars to increase extraction bed load (scalable for loading > 700 ng of cfDNA) and immobility buffer (IB) containing salts and polyethylene glycol to create cfDNA density on the activated plastic chip surface. More than 90% purity of cfDNA extraction was observed. Also, the IB buffer led to a reduction in coextracted DNA interference in the final results. The chip can also be used to detect clinical diseases such as KRAS mutations using cfDNA extracted from plasma specimens of patients with non-small-cell lung and colorectal cancer [88, 93]. Aravanis et al. demonstrated a novel method for the detection of ctDNA in patients’ plasma with colorectal cancer. They utilized microwells and a microfluidic platform based on microcolumns and, using dimethyl dithiobispropionimidate (DTBP), isolated ctDNA. In the following, using the Sanger sequencing technique, the ctDNA was verified. This study reported that DTBP drastically reduced the recognition of cellular backgrounds, especially DNA, which may be released from non-cancerous cells during the process of chemical mixing or washing. This study reported that DTBP drastically reduces the recognition of cellular backgrounds, especially DNA, which may be released from non-cancerous cells during the process of chemical mixing or washing. DTBP is used as the elution buffer, which binds to the amine groups of ctDNA, while sodium bicarbonate is used as the isolating agent. This method detects 71.4% of BRAF and KRAS mutation profiles in stages I-IV of disease in patients with colorectal cancer in 15 min [94]. The development of cheap and fast CRISPR-based nucleic acid detection methods may help the early detection of cancer [95]. In prokaryotes, CRISPR systems are complex with their effectors (mainly Cas12 or Cas13 proteins) and guided by a CRISPR-RNA (crRNA) for targeting and cleaving the nucleic acids of pathogens [96, 97]. In this regard, Zhang et al. introduced the Specific High-Sensitivity Enzymatic Reporter UnLOCKing (SHERLOCK) method. Combining recombinase polymerase amplification (RPA) technology with collateral ssDNase activity of Cas13, utilized in this method, is used for highly sensitive and specific detection of mutations associated with cancer in cfDNA, such as EGFR-T790M and EGFR-L858R mutations in patients with non-small-cell lung carcinoma (NSCLC) [98]. For this purpose, after performing a liquid biopsy, the intended mutations in cell-free DNA are detected by fluorescent and lateral flow-based readout methods (Fig. 2) [99].

In the last decade, the use of circulating cell-free tumor RNAs (ctRNAs) obtained from fluid biopsy, including mRNAs and non-coding RNAs (ncRNAs) such as miRNAs and long non-coding RNAs (lncRNAs), has been considered as biomarkers for the diagnosis of cancer [100]. Currently, techniques used for body fluid-derived ctRNA analysis include quantitative polymerase chain reaction (qPCR), next-generation sequencing, and digital drip PCR (ddPCR) [101]. The utilization of these methods in laboratories is limited due to their high cost and the need for developed laboratory equipment, trained labor, and time-consuming sample preparation. Thus, rapid and inexpensive diagnostic tools can enable the wider use of fluid biopsy analysis [95]. The CRISPR-Cas13 system is one of the new methods for cancer diagnosis. Cas13, which differs from other CRISPR-related enzymes in that it targets single-stranded RNA (ssRNA), can be used to detect RNA without the need for cDNA synthesis or nucleic acid amplification [102]. Tian et al. based on the Cas13-based RNA detection method divided Cas13/crRNA complexes, RNA targets, and fluorescent reporter ssRNAs into thousands of picoliter-sized droplets on a microfluidic chip for increasing concentrations of target RNAs and sensitivity. Then, in order to digitally quantify the target RNAs, fluorescence microscopy was employed to count positive droplets. This method successfully detected various RNA types, such as overexpressed circulating miRNAs in cancer. Using this technique, they showed a several-fold increase in miR-17 in breast adenocarcinoma and glioma cell lines and in miR-21 in patients with breast cancer [103]. According to a different study, a Cas13-based electrochemical microfluidic biosensor was developed by Bruch et al. for the detection of two miRNAs (miR-19b and miR-20) upregulated in patients with medulloblastoma [104]. In this method, activated Cas13 by miRNA binding cleaves reporter ssRNAs labeled with FAM, which leads to the removal of glucose oxidase labeled with anti-FAM antibodies. In the absence of miRNAs in the specimen, H2O2 was produced by glucose oxidase and detected in the electrochemical cell. Thus, the presence of targets of miRNA in the specimen reduces the signal [105]. In order to diagnose NSCLC in its early stages, Seng et al. designed an electrochemical biosensor chip based on CRISPR-Cas13 to detect multiple circulating RNAs. For the detection of EGFR and TTF-1 mRNAs along with miR-17, miR-19b, miR-155, and miR-210, Cas13 trans-cleavage activity was used for triggering the catalytic hairpin assay and ultimately signal generation. A designed chip like this can be used 37 times continuously for RNAs detection without sensitivity reduction. In this study, subjects were divided into four groups, including 30 healthy individuals, 12 patients with benign lung disease, 20 NSCLC patients with stage I-II, and 55 patients with stage III − IV. The sensitivity and specificity of the biosensor for distinguishing non-cancerous groups from cancer groups with different stages were 97.3 and 95.2%, respectively, while in the analysis between healthy subjects and early-stage cancer patient groups, they were 90% and 95.2%, respectively. As demonstrated by this study, such biosensors are highly sensitive and efficient for the simultaneous detection of RNA markers [106].

## In vitro microfluidic models of tumor microenvironment

After tumor formation, tumor cell signaling causes various significant changes at cellular and molecular levels. These observed modifications lead to the creation of a complex environment that includes stromal cells, immune cells, and an extracellular matrix (ECM) called the tumor microenvironment (TME) [107, 108]. The crucial function of TME in tumor growth and progression has been well understood, making it a major target for cancer treatment. Therefore, studies about the relationship between tumor cells and TME components open up new perspectives on molecular mechanisms that promote the development, progression, and metastasis of tumors and can also be effective in developing advanced therapies [109]. Among the cell culture methods, three-dimensional (3D) cultures can reconstitute tumor cells and TME biochemical components more realistically than two-dimensional (2D) cultures. Furthermore, results obtained from 3D in vitro models demonstrate a valid correlation with clinical findings [110–112]. Microfluidic devices, which can induce microenvironmental tumor signals by producing oxygen, reagents, and fluid pressure gradients with a high degree of time and spatial control, are one of the newest techniques for studying dynamic 3D microenvironments [113, 114]. In 2020, researchers cultured the macrophages and human ovarian adenocarcinoma cells (SKOV3) tumor spheroids into a 3D gel matrix simultaneously and designed a tumor-microenvironment-on-a-chip (TMOC) device to investigate the migration and interaction of macrophage-based drug carriers with the TME, utilizing drug-loaded macrophages. The developed TMOC model provided real-time monitoring of macrophage migration and infiltration into the tumor and showed that drug-carrying macrophages had a considerable ability to penetrate the tumor and cause high toxicity in tumor cells compared to other nanoparticle drug carriers. It has been suggested that macrophages are promising therapeutic tools for targeted drug delivery, and TMOC is a multi-purpose platform for rapid assessment of these drug delivery methods compared to complicated clinical investigations [115]. Ahn, Lim, et al. created a microfluidic platform in 2019 to study the communication between the TME and hydroxyapatite (HA) by culturing gastric cancer (MKN74) and colorectal cancer (SW620) cells into the HA and fibrin. HA is the main constituent of the bone extracellular matrix and influences several cellular responses in the TME that are associated with bone metastasis. Analysis indicated that a TME rich in HA can alter tumor cells' cytoplasmic quantity, cell viability, and proliferation. A higher concentration of HA can also inhibit the migration of tumor cells and cause fewer angiogenic sprouts, which are provoked by TME secreted paracrine factors. The designed microfluidic device facilitated the uncomplicated development of 3D TME, which is made of hydrogel and various cell types and enabled the monitoring of the HA concentration and culture time that affects the TME, which is a beneficial platform for drug screening and mechanical studies of bone tumor metastasis [116]. In another study, an in vitro microfluidic platform was developed to understand molecular and cellular principles for tumor–stroma communications and stroma during cancer invasion thru co-culture of fibroblast and breast cancer cells on a chip that mimics TME structure accurately. Through a combination of functional estimations and transcriptome profiling, it was found that invasion in breast cancer cells was facilitated by cancer-associated fibroblasts (CAFs). The 3D microfluidic coculture device in this study included tumor and stroma regions to simulate the structure of the early TME. The spatial design facilitated the two-way cross-talk between the tumor cells and the stromal cells, while still providing accurate monitoring [117]. In 2021, Surendran and colleagues developed a novel in vitro tumor immune microenvironment (TIME)-on-chip device to study the influence of neutrophils on the invasion initiation in ovarian tumor cells. For this purpose, tumor spheroids are cultured within hydrogel-based multi-microwell plates and embedded in a collagen matrix integrated by 3D bioprinting, which is regenerated in vitro to display neutrophil migration and tumor invasion. The results confirmed the ability of the microfluidic device to facilitate 3D interstitial fluid transportation around the spheroids to maintain prolonged cultures [118].

## Microfluidic approaches to the study of angiogenesis and anti-angiogenesis

**Fig. 3:**
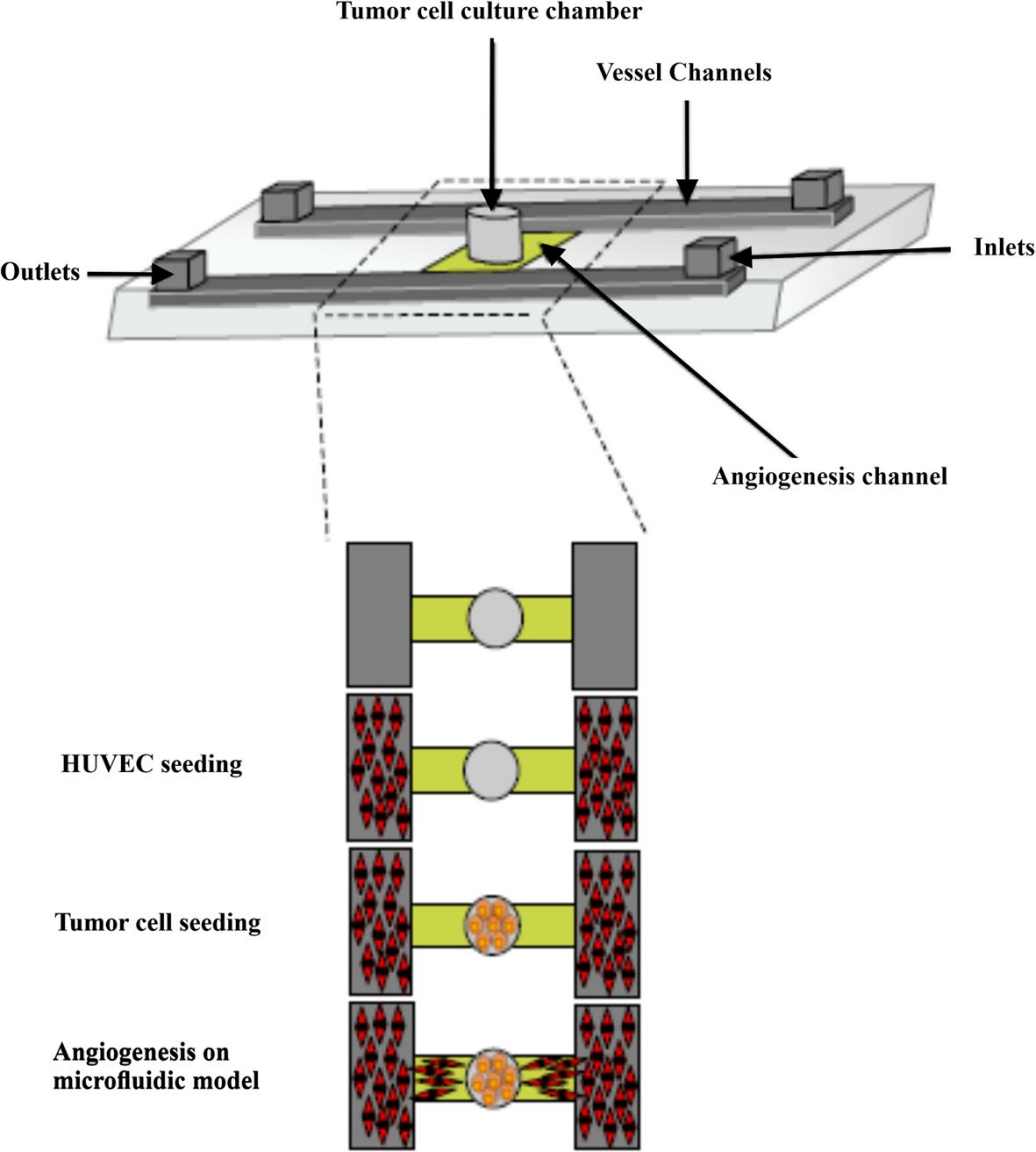
Biomimetic tumour-induced angiogenesis in a microfluidic device. The angiogenesis unit is depicted in this diagram, which has one open cell culture chamber and two channels of angiogenesis. The steps of cell loading are depicted in the diagram

When compared to typical in-vitro methods for angiogenesis and anti-angiogenic drug screening, in-vitro 3D microfluidic systems present a number of advantages [119–122]. Cancer-induced angiogenesis reproduced in a microfluidic model has been demonstrated to have various benefits, including boosting cell culture capacity and providing a time-saving, affordable, and rapid alternative to animal models [123]. Furthermore, microfluidic devices significantly reduced cell consumption compared to standard 2D culture systems, allowing the use of restricted primary cells from cancer patients in future investigations and offering the potential to screen therapeutic attitudes for individual patients in vitro (Fig. 3) [124].

Some anti angiogenic medicines, like Thalidomide, have been found to cause teratogenic consequences. The existence of chiral carbons could be to blame for the teratogenic effects. Annalisa Mercurio and colleagues produced four distinct phthalimide derivatives as Thalidomide analogs without chiral carbons in their general chemical configuration in 2019. They used an in-vitro 3D microfluidic experiment with human endothelial cells to look at their anti-angiogenic effects. When compared to Thalidomide, all four derivatives caused significant suppression of neovascularization at lower effective doses. They also emphasized the use of an in vitro 3D model for quick drug analysis and the selection of novel and more secure medications [125]. Liu et al. created a microfluidic system to test the impact of antiangiogenic medicines on angiogenesis generated by adenoid cystic carcinoma (ACC) and oral squamous cell carcinoma (SCC). The anti-VEGF effect on angiogenesis was studied in microfluidic and nude mouse models, and it was found that it successfully stopped the process in both microfluidic and nude mouse models [126, 127]. Lim et al. created a three-dimensional in vitro model for HCC micro-vascularization regeneration in hypoxia. The platform allows researchers to examine the expression, proliferation, angiogenesis, and drug resistance of hypoxia-inducible factor 1 (HIF-1) in hypoxic and microvascularized HCC after culturing and drug screening. The results indicate that using this microfluidic system to explore hypoxic HCC with microvasculature could lead to the development of anti-cancer therapies [128]. Vincent van Duinen and colleagues developed a perfused 3D angiogenesis examination on endothelial cells (ECs) derived from induced pluripotent stem cells (iPSCs) in 2020 and evaluated its implementation and appropriateness for anti-angiogenic drug analysis. The results suggest a robust and scalable assay that includes physiologically relevant culture conditions and is amenable to the screening of anti-angiogenic compounds [129].

### RNA interference (RNAi) to the study of angiogenesis and anti-angiogenesis

One of the most extensively utilized molecular tools in functional genomics investigations is RNAi, which is a sequence-specific gene silencing strategy. Exogenous double-stranded RNA (dsRNA) or small interfering RNA (siRNA) is used in RNAi to hinder protein production by inhibiting the expression of the appropriate mRNA [130]. The appropriate assessment of RNAi-based anti-angiogenic nanoscale medicine requires three-dimensional visualization of tumour vasculature as a crucial consideration. Yet, due to the lack of an adequate physiological in-vitro model or exact analytic procedure, this remains a challenge. To overcome this constraint, Lee and colleagues constructed a 3D microfluidic device to mimic effective 3D angiogenic sprouting cultured simultaneously with different types of cancer cells. This model allowed for a quick and effective evaluation of anti-angiogenic nanomedicine in the case of a hyper-vascularized tumour. They also discovered tumour sub-regional differences in anti-angiogenic efficacy utilizing this 3D imaging-based approach [131]. In 2017, Xueqin Huang et al. used a microfluidic hydrodynamic focusing (MF) tool to create polymer-lipid hybrid nanoparticles (P/LNPs) loaded with VEGF siRNA. The P/LNPs synthesized by MF hampered tumour cells in vitro while causing minimal cytotoxicity. In a xenograft tumour model, P/LNPs generated with microfluidic technology showed higher down-regulation of VEGF mRNA and protein expression and also more tumour neovessel blockage. P/LNPs encapsulating VEGF siRNA produced using the MF technique (P/LNPs-siRNA-MF) is an effective therapeutic siRNA delivery system for cancer therapy either in-vitro or in-vivo [132].

## Microfluidic platforms for the study of cancer metastasis

A series of cellular and molecular processes results in cancer spreading to other parts of the body, which is called metastasis. Beginning with the spread of preliminary tumor cells that experience metamorphosis and develop aggressive characteristics such as the capability to migrate and penetrate the extracellular matrix, culminating in the production of secondary tumors [133, 134]. In a nutshell, it is a complicated, multi-phased procedure that begins with the escaping of cancer cells from tumor tissue, known as epithelial-mesenchymal transition (EMT), followed by intravasation, staying alive in the blood circulation, migration of cancer cells to various sites throughout the body, extravasation and finally metastatic foci formation [133, 135–137]. In fact, the connection between cancer cells and the microenvironment of the tumour is the reason for this aggressive and invasive procedure, which becomes obvious when the tumour microenvironment is changed, caused by the onset of the disorder and advancement to the invasion phases [16, 135]. Zhang et al. developed a worm-based (WB) microfluidic device that can quickly track biochemical signals linked to metastasis in a controlled environment. Caenorhabditis elegans were put in a WB biosensor and treated with samples conditioned with cancer cell clusters in this experiment. The chemotactic preferences of the worms were followed by non-continuous imaging to reduce the effect on normal physiological function. To standardize quantitative analysis by the WB biosensor, a chemotaxis index (CI) was developed, with moderate and high CI concentrations indicating higher levels of metastatic threat and presence, respectively. The released metabolite glutamate was discovered to be a chemorepellent, and more extensive clusters associated with the higher metastatic possibility also elevated CI levels [138]. Cancer metastatic risk is linked to the epithelial-to-mesenchymal transition index. Tumor-derived extracellular vesicles (EVs) can be used to create the EMT index, which is a valuable tool for estimating metastatic chance. To create an EVs-based EMT index, each epithelial cell and mesenchymal cell-derived EV must be isolated separately. Hogyeong Gwak and colleagues developed a unique microfluidic tool for isolating two types of EVs in 2021. It was found that above 90% of the EVs representing both the epithelial marker (EpCAM) and mesenchymal marker (CD49f) could be divided eclectically per 100 µl of the sample volume in just 6.7 min [139]. Cho, Choi et al. developed a microfluidic platform to mimic the structure of lymph vessel-tissue-blood vessel (LTB) to realize the impacts of pro-inflammatory cytokines on lymphatic metastasis. The findings confirmed that during lymphatic metastasis, one of the inflammatory cytokines, interleukin 6 (IL-6) mediated intercellular interactions in the TME, induced EMT, and increased tissue invasion potential. The suggested LTB chip enables to utilize for analysis of intercellular interactions during metastasis and is a novel tool to realize intercellular interaction in the TME following different extracellular stimulations [140]. In another work, Mara and coworkers [141] dissected the role of the Cdk5/Tln1/FAK axis in vascular adhesion, TEM, and early invasion in human breast cancer using three independent microfluidic vascular in vitro models. They discovered the structural role of Tln1 and FAK, but not their phosphorylation, which supports actin polymerization required for invadopodia formation, leading to 3D-matrix invasion. The lung colonization of breast cancer cells drastically declined when Tln1 and FAK were silenced, as well as when FAK was chemically blocked. In a novel approach, Samandari et al. presented a straightforward strategy for providing an independent and reusable microfluidic gradient generator to investigate cellular functions, including the migration and invasion of cancer cells in reaction to chemical stimulants. By adjusting the PDMS treatment period to improve bonding strength, the PDMS was loaded directly onto commercial polystyrene-based cell culture surfaces by manipulating the PDMS curing time to optimize bonding strength. By minimizing either surface treatment or coating, the stand-alone technique not only allows for pumpless installation of this microfluidic device, but also guarantees limited fluidic force and, as a result, a leak-free system [142]. For quite some time, the *Dendropanaxmorbifera* plant has been used as a folk medicine to cure a wide range of diseases. Kim et al., 2020, used a cancer metastasis model evolved from a 3D microfluidic device to study the antimetastatic impacts of D. morbifera sap-derived extracellular vesicles (DMS-EVs). They discovered that DMS-EVs inhibited CAFs, which play an important role in cancer metastasis, in a concentration-dependent manner. A number of genes associated with growth factors and extracellular matrix, such as integrin and collagen, were also altered by DMS-EVs [143].

## Human organ-on-chip in cancer research

The development of 3D cancer spheroids and organoid models, despite their advantages over 2D culture to mimic the cancerous and normal tissues, still has some limitations, including a different tissue and cell interactions, vascular perfusion, physical and mechanical forces, and organ-level structures that exist in living organisms [144]. In addition, animal in vivo models couldn’t recapitulate human organ microenvironments [145]. Recent advances in microfluidic device technology have led to the development of human organs-on-chip (OOC) models that construct tissue and organ functionality levels not achievable with traditional culture methods [146]. Microfluidic devices designed by microchip manufacturing contain multichannel and tissue compartments to mimic the normal or diseased physiological and biological responses of organs [147]. While whole organ modeling is still challenging at this level, most OOC devices aim to mimic the crucial functions of tissues and organs to achieve specific applications [148]. Cancer-on-a-chip is an OOC model that can partially reproduce complex tumor microenvironments by replacing healthy cells and related extracellular matrix with those of cancer sources and can be utilized to investigate the interaction between cancer and other organs. Personalized medicine, high-throughput drug screening, drug efficiency assessment and metastasis studies are some of the applications of OOC platforms in cancer research [149]. Different cancer types, such as lung, bone, breast, prostate, liver, and colorectal cancer, have been developed in OOC devices to investigate crucial cancer biological processes [145]. Some of these platforms are detailed in Table 1. Moreover, cross-organ communication in cancer can cause metastasis, which is responsible for over 90% of cancer mortality. To understand the complex metastatic pathophysiological processes and to develop unique therapies, multi-organ-on-chip models that integrate the potential metastatic niches and tumors are highly desired [135, 150, 151]. Recently, a multi-organ on-chip was developed for real-time monitoring of the brain metastasis process, which cannot be achieved by other developed models. The designed platform consists of a functional blood–brain barrier (BBB), a downstream brain organ unit that was comprised of two 300 μm vascular chambers, one of which was linked to the upstream part for metastatic tumor cell transportation, two vascular chambers with independent perfusion ability, and a syringe pump to mimic blood circulation.

**Table 1:** Summary of recent organ-on-chip platforms in cancer research

Cancer organs-on-chips models	Cell types	Research objectives	Results	reference
Lung cancer-on-chip	NCI-H1437	Cancer on-chip real-time monitoring and cytotoxicity evaluation of drug compounds	Real-time assessment rivaled that compare to docetaxel the increasing concentrations of doxorubicin caused higher cell death rate	[152]
Bone-on-a-chip	MC3T3-E1	Bone metastasis study of breast cancer cells	Observation of breast cancer bone colonization hallmarks that were previously confirmed only by in vivo experiments	[153]
Liver-on-a-chip	SK-BR	Investigating the roles of breast cancer-derived EVs in liver metastasis	Activation of liver sinusoidal endothelial cells by breast cancer-derived EVs caused endothelial to mesenchymal transition and vessel barriers destruction, higher TGFβ1 levels, and upregulation of fibronectin, which facilitates the adhesion of breast cancer cells to the liver microenvironment	[154]
Ahepatocellular carcinoma–bone metastasis-on-a-chip	HepG2, HCC	Analyze the anticancer effects of thymoquinone (TQ) free and encapsulated form on HCC metastasis	The longer period of the inhibitory effect of nanoparticle-encapsulated TQ	[155]
Lung cancer metastasis multi-organ on-chip	16HBE, A549, HUVEC, WI38, THP-1, HA-1800, Fob1.19, L-02	Mimicking the in vivo microenvironment of lung cancer metastasis	Damage of astrocytes, osteocytes, and hepatocytes after cancer cell metastasis and in-vivo experiments validates the performance of metastasis in the designed platform	[156]
Colorectal cancer-on-chip model	Caco2 C2BBe1,HUVEC	Studying the microenvironmental influence on intravasation	CRC-On-Chip provides a human-relevant model system to study early invasive events in cancer	[145]
Heart-breast cancer-on-a-chip	SK-BR-3, iPSC	Disease modeling and monitoring of cardiotoxicity induced by cancer chemotherapy	The platform will allow early detection and prediction of chemotherapy-induced cardiotoxicity in individual patients	[145]
Kidney cancer metastasis-on-a-chip	Caki-1, HepLL	Mimicking the progression of kidney cancer in the liver for predicting treatment efficacy	Platform indicating superior efficacy to free anti-cancer drug (5-FU) in killing Caki-1 cells and a linear anti-cancer relationship between the concentration of 5-FU and the percentage of Caki-1 cells	[157]
Lung cancer-on-a-chip	A549, HFL1, HUVEC	Investigating the impact of the interaction between tumor cells and fibroblasts on tumor invasion, metastasis, and drug resistance	A549 co-cultured with HFL1 cells indicating anti-cancer drug resistance, also A549 co-culture with HFL1 and HUVECs revealing that the A549 cells could induce apoptosis or death of endothelial cells	[158]
Heart/Liver cancer on a chip	HepG2, hCMs	Reproducing the side effects of anti-cancer drugs in vitro	Evaluating the cytotoxicity of an anti-cancer drug on cancer cells and normal cardiomyocytes within the device	[159]

**Fig. 4:**
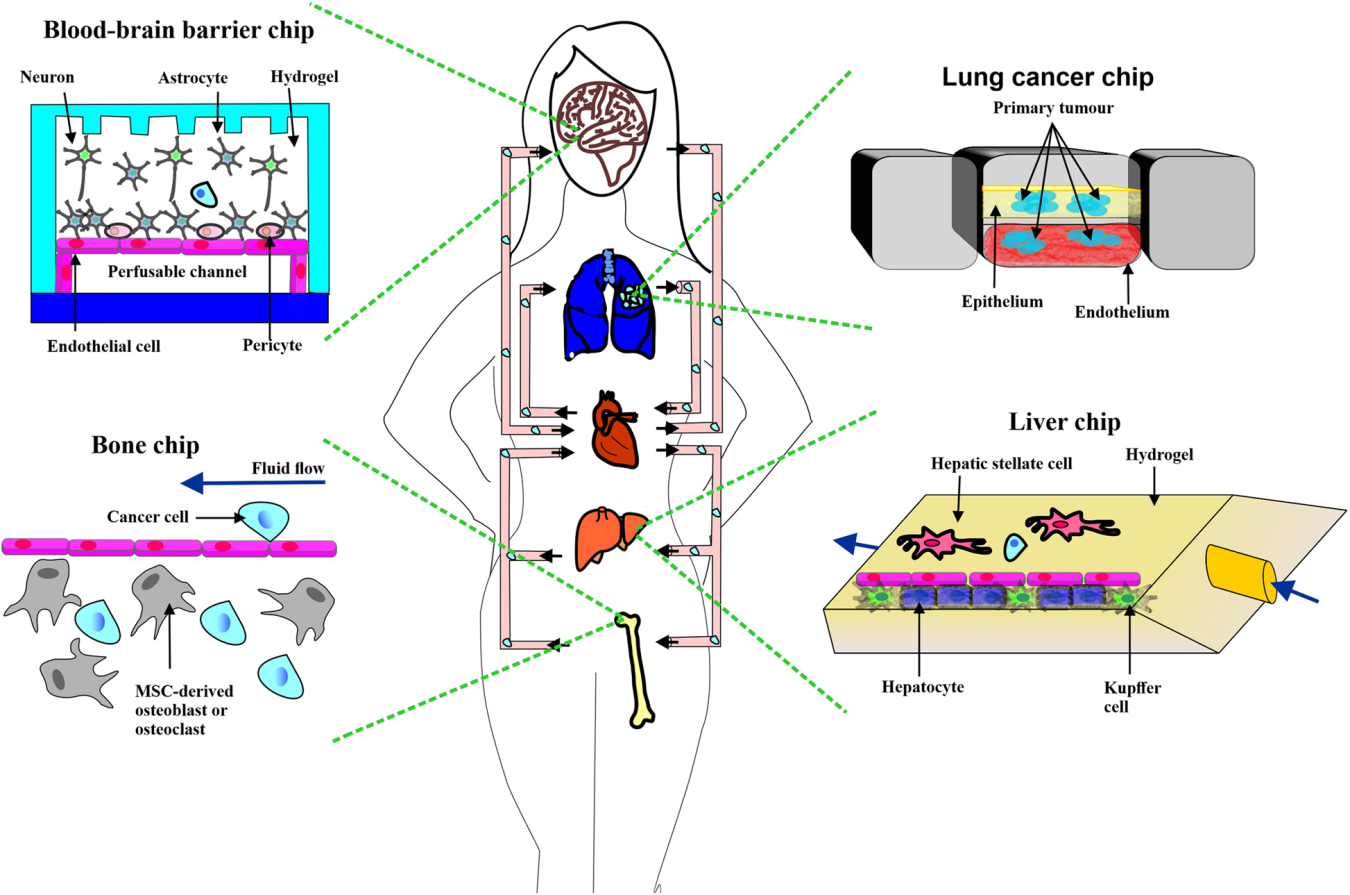
Modelling systemic metastasis in a body-on-chip An illustration is provided to represent the metastatic progression in the future using a human body-on-chip consisting of multiple fluidically connected organs-on-chips, which are often referred to as organ chips, such as the liver, brain, lung, and bone chips. On this body-on-chip, arrows indicate blood circulation, showing lung cancer cells growing on a lung cancer chip invading the vascular channel. Then cancer cells spread to the other chips, owing to fluid connections and pumping of the same medium to multiple chips. This is similar to how blood is pumped from the heart to every other organ in the body. The progression of metastatic lesions could be monitored by observing lung cancer cells with fluorescent markers penetrating the circulation of fluid. These markers could be inserted into the liver, bone, or brain chips from afar. Metastasizing lesions typically occur at these sites where studies could be conducted to identify and study the growth of metastatic cancer cells. By using this method, it would be possible to determine the mechanisms by which tumour cells attack particular organs (organotropism) and also recognize possible pharmacological approaches to inhibit metastatic cancer cells spread

Findings revealed that the expression of Aldo–keto reductase family 1 B10 was increased in lung cancer brain metastasis. A multi-organ-on-chip designed for the metastatic spread of cancer consists of liver, bone, lung, heart, and brain chips as illustrated in Fig. 4.

## Microfluidic techniques for cancer therapies

Additionally, it allows clinicians to carry out proper patient characterization even with insufficient raw materials [160–163]. Jeong, Yu et al. engineered a microfluidic device that is cocultured with NSCLC cells and endothelial cells to study the effects of the exosome-miRNA delivery system on lung cancer cells. They showed that lung tumor growth and angiogenesis were dramatically suppressed by miR-497 in comparison with controls, indicating that the exosome-miR delivery system coupled with the microfluidic platform is enabled to regulate the temporal and spatial conditions in the tumor microenvironment and is a cost-effective and accurate mimicry device for the improvement of targeted cancer therapy [164]. In the field of drug screening, Fu, Zuo, and colleagues designed a wick-like paper-based microfluidic platform with a newly developed folded paper tape as a wick-like construction, which was utilized for medium-automated perfusion. By adopting the suggested microfluidic tool, they used two types of hepatoma cancer cells (MCF-7 and HepG2) as model cells for cell culture and drug screening. By providing some advantages, such as an affordable price, ease of use, and miniaturization, they demonstrated that their microfluidic device can be highly efficient for anti-cancer drug screening [165]. In another study, Khot, Levenstein et al. developed a 3D microfluidic device by culturing cells into the chip to screen drugs for cytotoxic activity by treating the spheroids with 5-Fluorouracil as a chemotherapeutic drug for five days. Real-time cell viability was analyzed through fluorescence microscopy. Also, a Lactate dehydrogenase (LDH) assay was performed on the supernatant. Their findings revealed an increase in cell death, and they confirmed that their developed microfluidic device enables on-chip cancer therapy, quantitative and qualitative viability examination, and establishes the principle of an efficient device for convenient, fast, and high-throughput anti-cancer drug screening in complex tumor spheroids [163]. In a novel work, Chang, Jiang et al. created a biomimetic Metal–organic Nanoparticle (MON) formulation and developed a microfluidic device to further enhance the fabrication of MONs which could accurately regulate the nanoparticle development process and also has elevated potential for large-scale generation of nanoparticle formulations. The developed nanoparticle showed strong anti-cancer activities in the cancer cells and provided an innovative approach to producing a biomimetic nanoparticle formulation for cancer treatment [166].

## Advantages & challenges

As previously mentioned, microfluidic devices in comparison to conventional systems have various advantages in cancer therapy. Current methods for cancer therapeutics are mainly based on the use of cytotoxic agents and don’t specifically target cancer cells [167–169]. Therefore, other treatment methods are needed that can improve cancer therapies by targeting cancer cells specifically. Microfluidic systems are new methods of cancer diagnostics and therapeutics that have a high potential to improve treatment results. In addition, such methods are more suitable for easy use in cancer diagnosis than other common technologies [170]. These advantages compromise a reduction in drug and biological sample consumption, more accurate control of spatiotemporal parameters and fluids in the TME, real-time monitoring of cell interactions and invasion, reliable tumor and TME mimicking, and enhanced control of the environment [171–174]. In addition, tumor diverse populations show different responses to therapies, which can be a barrier for clinicians in cancer treatment. In vivo microfluidic systems enable the preservation of cancer cells' heterogeneity by presenting accurate in vivo TME and overcoming this obstacle [175]. Additionally, in adoptive cell-mediated cancer immunotherapies, 3D microfluidic tumor models can modulate cytokine delivery and superimpose chemokine gradients. Furthermore, since microfluidics can be seeded with patient-derived cells, personalized immunotherapeutic strategies can be identified to fight against cancer [176]. Among these remarkable advantages, there remain some limitations with microfluidic devices that can affect cancer therapy studies. PDMS, frequently utilized for microfluidic device fabrication, has some limitations such as toxicity caused by the gradual release of oligomers and molecules absorption. Also, physiologically appropriate matrix compositions and cell types are required to mimic the native TME. Moreover, current microfluidic devices need to be improved to better recapture the physiological complexity of the in vivo systems [176, 177]. High technology and sophisticated manufacturing processes required for making micrometer-scale structures, use of suitable materials in any microfluidic system considering the final application, mass production, commercialization of microfluidic devices, high experimental knowledge for the wide use of these systems in most laboratories, and fostering widespread acceptance of such technology in the diagnosis or treatment of cancer are substantial challenges that do not resolve overnight [178, 179]. As of now, most microfluidic devices are limited to quasi-2D planar formats, and the use of 3D printers to build microfluidic devices is being assessed. Current studies still have a strong technological rather than an application focus and are not compelling for the use of these systems as enabling tools [179].

## Conclusions

In recent decades, considerable developments have been recorded relating to microfluidic systems along with microfluidics-based tests. These platforms have been extensively employed in various fields related to cancer investigations such as drug screening and delivery, cancer detection, and using diverse approaches of treatment like gene therapy, chemotherapy, and radiation therapy. Microfluidics could make drug delivery systems in exceptional and controlled conditions and also assess the impacts of encapsulated drugs for early screening to guarantee their efficacy on cancer cells. Besides, microfluidics can emulate the characteristics of organ-on-a-chip, human organs body, and allow scientists to analyze the safety of novel therapeutic drugs prior to clinical use. Today, this technology lets researchers to detect cancer rapidly and inexpensively. Great precision in measuring certain diagnostic factors with a lesser sample size is one of the main benefits of this system, which has made it an unequaled competitor to common diagnostic tests.

## Data Availability

Not applicable.

## Abbreviations

CTCs: Circulating Tumor Cells
CAGR: Compound Annual Growth Rate
ASIC: Application-specific Integrated Circuits
PDMS: Polydimethylsiloxane
PCR: Polymerase Chain Reaction
TRL: Technology Readiness Level
POC: Point-of-care
ELISA: Enzyme-linked Immunosorbent Assay
GaN: Gallium Nitride
HEMT: High Electron Mobility Transistor
FET: Field Effect Transistor
SERS: Surface-enhanced Raman Spectroscopy
FRET: Fluorescent Resonance Transfer
EpCAM: Epithelial Cell Adhesion Molecule
RIfS: Reflection Interference Spectroscopy
AAO: Anodic Aluminum Oxide
PLGA: Glycolic Acid-Lactic Acid
miRNAs: Micro RNAs
UC: Ultra-centrifugation
ctDNA: Circulating tumor DNA
cfDNA: Cell-free DNA
NGS: Next-generation Sequencing
SPμE: Solid-phase Microextraction
IB: Immobility Buffer
DTBP: Dimethyl Dithiobispropionimidate
crRNA: CRISPR-RNA
SHERLOCK: Specific High-Sensitivity Enzymatic Reporter UnLOCKing
RPA: Recombinase polymerase amplification
ctRNAs: Cell-free tumor RNAs
NSCLC: Non-small-cell lung carcinoma
ncRNAs: Non-coding RNAs
lncRNAs: Long ncRNAs
qPCR: Quantitative polymerase chain reaction
ddPCR: Digital drip PCR
ssRNA: Single-stranded RNAs
ECM: Extracellular matrix
TME: Tumor microenvironment
3D: Three-dimensional
2D: Two-dimensional
SKOV3: Macrophages and human ovarian adenocarcinoma cells
TMOC: Tumor-microenvironment-on-a-chip
HA: Hydroxyapatite
CAFs: Cancer-associated Fibroblasts
TIME: Tumor Immune Microenvironment
ACC: Adenoid Cystic Carcinoma
SCC: Squamous cell carcinoma
HIF-1: Hypoxia-inducible factor 1
ECs: Endothelial cells
iPSCs: Induced pluripotent stem cells
RNAi: RNA interference
dsRNA: Double-stranded RNA
siRNA: Small interfering RNA
MF: Microfluidic hydrodynamic Focusing
P/LNPs: Polymer-lipid Hybrid Nanoparticles
EMT: Epithelial-Mesenchymal Transition
WB: Worm-Based
CI: Chemotaxis Index
EVs: Extracellular Vesicles
LTB: Lymph vessel-tissue-blood vessel
IL-6: Interleukin 6
DMS-EVs: D. Morbifera Sap-derived Extracellular Vesicles
OOC: Organs-On-Chip
BBB: Blood–Brain Barrier
TQ: Thymoquinone
LDH: Lactate Dehydrogenase
MON: Metal–organic Nanoparticle
